# The relationship between the Prognostic Nutritional Index and lymphovascular and perineural invasion of the tumor in patients diagnosed with gastric cancer, and its effect on overall survival

**DOI:** 10.1097/MD.0000000000040087

**Published:** 2024-10-18

**Authors:** Pırıltı Özcan, Mehmet Sinan Çarkman

**Affiliations:** a Department of General Surgery, Istanbul University, Cerrahpaşa Faculty of Medicine, Istanbul, Turkey.

**Keywords:** gastric cancer, lymphovascular invasion, perineural invasion, Prognostic Nutritional Index

## Abstract

A low Prognostic Nutritional Index (PNI) value, lymphovascular invasion (LVI), and perineural invasion (PeNI) have been identified as indicators of poor prognosis for many malignancies. We aimed to evaluate the relationship between PNI and LVI/PeNI, their prognostic significance, and their effect on overall survival in gastric cancer patients who underwent curative gastrectomy. A cutoff value of 39.8 was taken for the PNI, and PNI < 39.8 was defined as moderate to severe malnutrition. Patients were grouped as PNI-low (PNI < 39.8) and PNI-high (PNI ≥ 39.8). Paraffin-embedded tissue sections of surgical specimens were used to evaluate PeNI as defined by previously reported criteria. The study included 270 patients with ages ranging from 23 to 90 years. The mean PNI was calculated as 39.8 ± 6.35. PeNI was detected in 232 patients (85.93%), and LVI was identified in 248 patients (91.85%). It was observed that the PNI value of patients with an expired status in the PNI < 39.8 group was lower compared to those who survived, and in patients with PNI > 39.8, those without PeNI had better survival. The presence of PeNI in patients with PNI > 39.8 increased the mortality risk by 2.088 units, while in patients with PNI > 39.8, it was found that those without LVI had better survival, and the presence of LVI increased the mortality risk by 3.171 units. Mortality developed in 166 patients (61.48%) during the five-year follow-up period. The five-year overall survival was found to be 31.02 ± 21.73 months. In patients with gastric cancer, the PNI, LVI, and PeNI are independent prognostic factors for overall survival in postoperative patients. A low PNI score is an inherently poor prognostic factor. In patients with a high PNI score, the presence of positive LVI and PeNI negatively impacts survival. We found that in patients with a low PNI, the rates of PeNI and LVI are higher compared to those with a high PNI, and this significantly affects mortality.

## 1. Introduction

Gastric cancer (GC) is the 5th most common malignancy worldwide and ranks 4th in cancer-related deaths.^[1]^ It has the highest incidence and mortality rates, particularly in Far East and East Asian countries. Multiple multifactorial elements can play a role in the etiology of GC. These include microbial agents like *Helicobacter pylori*, environmental factors such as smoking, consumption of smoked and processed foods, high-salt diets, and limited intake of fruits and vegetables. Common presenting complaints of GC include weight loss, persistent abdominal pain, dysphagia, hematemesis, and early satiety. Early-stage GCs are often asymptomatic, which underscores the importance of gastroscopy in screening and diagnosis. During gastroscopy, biopsies of suspicious lesions can confirm the diagnosis of GC. The depth of tumor invasion, the number of regional lymph node metastases (LNM), and distant metastases are crucial for predicting overall survival in GC. GC is typically diagnosed at an advanced stage with a poor prognosis, where LNM significantly impacts prognosis and guides clinical management.^[2,3]^

The Prognostic Nutritional Index (PNI) has been used as a prognostic indicator for GC due to its efficacy and ease of calculation.^[4–6]^ It is widely employed to preoperatively assess patients with gastrointestinal malignancies and predict surgical risks.^[7–9]^ Lymphovascular invasion (LVI) is histologically defined by the presence of tumor cells within lymphatic or vascular channels or by the destruction of lymphatic or vascular walls by cancer cells. Perineural invasion (PeNI) is identified histologically by the invasion of nerve structures by the tumor and its spread along nerve sheaths.^[10,11]^ A low PNI value, LVI, and PeNI have been identified as indicators of poor prognosis in many malignancies. Literature reports indicate that the prevalence of low PNI and LVI/PeNI is relatively high in GC compared to other cancer types such as colorectal cancer (CRC) and esophageal squamous cell carcinoma.^[12–19]^ However, while the prognostic value of PNI in GC has been highlighted by recent studies, the prognostic significance of LVI and PeNI remains controversial. Some researchers have found LVI and PeNI to be independent risk factors for survival outcomes in GC patients.^[20–23]^ Others have determined that although LVI and PeNI are strongly associated with disease progression in GC, they are not independent prognostic factors.^[24–29]^

In this study, we hypothesized that PNI is related to LVI/PeNI and the survival outcomes of GC patients. We aimed to evaluate the relationship between PNI and LVI/PeNI, their prognostic significance, and their effect on overall survival in GC patients who underwent curative gastrectomy.

## 2. Materials and methods

### 2.1. Data collection

Data from 270 patients diagnosed with gastric adenocarcinoma who underwent total/subtotal gastrectomy at the Department of General Surgery, Istanbul University-Cerrahpaşa, Cerrahpaşa Medical Faculty, between August 2016 and February 2023 were retrospectively analyzed. Demographic data, including gender (female and male), age, preoperative lymphocyte counts, albumin levels, approximate tumor size (≤5 and >5 cm), tumor differentiation (well, moderate, and poor), Lauren classification (intestinal, diffuse, and indeterminate), depth of infiltration (T1, T2, T3, and T4), lymph node status (N0, N1, N2, and N3), TNM stage (I, II, and III), PNI, PeNI (negative and positive), LVI (negative and positive), morbidity, and mortality data were obtained from the hospital information system and patient files. The study commenced after obtaining ethical approval (2023/786077) from the Cerrahpaşa Medical Faculty Ethics Committee. Patients who are over 18 years old diagnosed with gastric adenocarcinoma, who had not received neoadjuvant therapy and had no distant organ metastasis and/or negative peritoneal cytology, no diagnosis of a second primary cancer, had at least 16 lymph nodes excised in the pathology result, and underwent elective surgical operation were included in our study. Patients who are operated for benign reasons, stage 4 patients with distant metastasis, emergency cases and pathological reports other than gastric adenocarcinoma (gastric lymphoma, neuroendocrine tumor, and /) and patients with incomplete data were excluded from our study. Preoperative clinical staging was based on gastroscopy and CT scans.

### 2.2. Prognostic Nutritional Index (PNI)

All laboratory data used to calculate preoperative nutritional parameters were obtained within 1 week before surgery. PNI was calculated using the following formula: 10 × serum albumin concentration (g/dL) + 0.005 × lymphocyte count (number/mm³). In this study, the cutoff value for PNI was set at 39.8, with PNI < 39.8 defined as moderate to severe malnutrition. Patients were grouped into PNI-low (PNI < 39.8) and PNI-high (PNI ≥ 39.8).

### 2.3. Perineural invasion

Paraffin-embedded tissue sections from surgical specimens were used to evaluate PeNI, defined according to previously reported criteria. Immunohistochemistry staining for the S100 protein, which is specific to nerves, was used to aid in the detection of nerves within gastric tissues. Hematoxylin and eosin staining was used to confirm nerve structures. Samples where PeNI was observed were classified as PeNI-positive.

### 2.4. Lymphovascular invasion

LVI was defined as the invasion of tumor cells into the vessel walls and/or the presence of tumor emboli within an endothelial-lined space. No distinction was made between vascular and lymphatic vessels. The diagnosis of LVI was made using the 8th edition of the TNM classification for malignant tumors.

### 2.5. Statistical analysis

Statistical analyses were performed using SPSS version 25.0 (Chicago, IL). The normality of the distribution of variables was assessed using histogram graphs and the Kolmogorov–Smirnov test. Descriptive analyses were presented using mean, standard deviation, median, and minimum–maximum values. For nonparametric variables that did not show a normal distribution, the Mann–Whitney *U* test was used to compare 2 groups, while the Kruskal–Wallis test was used for comparisons among more than 2 groups. The relationship between measured variables was analyzed using the Spearman correlation test. The significant cutoff value of PNI that could predict mortality was examined using ROC analysis. Kaplan–Meier test and Cox Regression Analysis were used for survival analyses. The impact of PNI on mortality was determined using Binary Logistic Regression analysis. A *P*-value of <.05 was considered statistically significant.

## 3. Results

Our study consisted of 270 patients (186 male and 84 female). The mean age was 61.45 ± 12.27 years. The ages of the patients ranged from 23 to 90. The mean lymphocyte and albumin values were determined to be 1.76 ± 0.64 and 3.98 ± 0.63, respectively. When examined in terms of GC localization, it was observed that 97 (35.93%) originated from the cardia, 88 (32.59%) from the corpus, and 85 (31.48%) from the antrum. The PNI average was calculated as 39.8 ± 6.35. PeNI was found positive in 232 cases (85.93%), and LVI was positive in 248 cases (91.85%). Subtotal gastrectomy was performed on 56 cases (20.74%) (1 laparoscopic and 55 open procedures), while total gastrectomy was performed on 214 cases (79.25%) (8 laparoscopic and 206 open procedures). Additional organ resections were performed on 46 cases (17.03%). All cases underwent D2 lymph node dissection. Of the 261 cases, 251 (96.7%) underwent open surgery, while 9 (3.3%) underwent minimally invasive surgery (laparoscopic and robotic). The mean operation duration was 165 minutes (ranging from 80–310 minutes). Intensive care unit admission was required for 59 cases (21.85%), while 211 cases (78.15%) were transferred to the regular ward postoperatively. According to histological classification (World Health Organization [WHO]), adenocarcinoma was detected in 201 cases (74.44%), signet ring cell carcinoma in 64 cases (23.7%), mixed adenocarcinoma in 3 cases (1.11%), and mucinous adenocarcinoma in 2 cases (0.74%).

The mean tumor diameter was found to be 6.84 ± 4.39 cm, and the number of regional lymph nodes was 28.40 ± 11.83. Regional lymph node involvement was present in 188 cases (74.44%). Regarding invasion depth, T1 tumors were found in 33 cases (12.22%), T2 in 17 cases (6.30%), T3 in 41 cases (15.19%), and T4 in 179 cases (66.30%). In terms of N stage classification, N0 involvement was observed in 69 cases (25.56%), N1 in 32 cases (11.85%), N2 in 52 cases (19.26%), and N3 in 117 cases (43.33%). According to the pathological TNM staging based on the American Joint Committee on Cancer staging system, the numbers of cases classified as stage I, II, and III were determined to be 43 (15.93%), 45 (16.67%), and 182 (67.41%), respectively. Mortality occurred in the first 30 days in 2.2% of cases. Over a five-year follow-up period, mortality developed in 166 cases (61.48%). The five-year overall survival was found to be 31.02 ± 21.73 months (Table 1).

**Table 1:** Demographic data.

		n/Mean ± SD	%/Median (Min–Max)
Age		61.45 ± 12.27	63 (23–90)
Sex	Male	186	(68.89)
	Female	84	(31.11)
Tumor localization	Antrum	85	(31.48)
	Cardia	97	(35.93)
	Corpus	88	(32.59)
Lymphocyte *%*		1.76 ± 0.64	1.7 (0.2–3.8)
Albumin (g/dL)		3.98 ± 0.63	4.1 (1.22–5.26)
Resection Type	Distal subtotal gastrectomy	47	(17.41)
	Distal subtotal gastrectomy + additional organ resection	8	(2.96)
	Conversion total gastrectomy	2	(0.74)
	Lap. distal subtotal gastrectomy	1	(0.37)
	Lap. total gastrectomy	7	(2.59)
	Lap. total gastrectomy + additional organ resection	1	(0.37)
	Total gastrectomy	167	(61.85)
	Total gastrectomy + additional organ resection	37	(13.70)
Microscopic type (WHO)	Adenocarcinoma	201	(74.44)
	Signet ring cell carcinoma	64	(23.70)
	Mixed adenocarcinoma	3	(1.11)
	Mucinous adenocarcinoma	2	(0.74)
Tumor size (cm)		6.84 ± 4.39	6 (0–28)
Number of regional lymph nodes		28.40 ± 11.83	25 (16–90)
Regional lymph node involvement	None	82	(30.37)
	Positive	188	(69.63)
Invasion depth	T1	33	(12.22)
	T2	17	(6.30)
	T3	41	(15.19)
	T4	179	(66.30)
N stage	N0	69	(25.56)
	N1	32	(11.85)
	N2	52	(19.26)
	N3	117	(43.33)
TNM stage	I	43	(15.93)
	II	45	(16.67)
	III	182	(67.41)
Prognostic Nutritional Index		39.8 ± 6.35	41.01 (12.21–52.61)
Perineural invasion	None	38	(14.07)
	Positive	232	(85.93)
Lymphovascular invasion	None	22	(8.15)
	Positive	248	(91.85)
ICU admission	None	211	(78.15)
	Positive	59	(21.85)
Mortality	None	104	(38.52)
	Positive	166	(61.48)
The day between surgery and death		567.08 ± 494.1	396 (0–1984)
Follow-up period months (5 years)		31.02 ± 21.73	26.21 (0–60)

Data are presented as median (interquartile range) or n (%).

ICU = intensive care unit, Lap = laparoscopic, TNM = tumor node metastasis, WHO = World Health Organization.

The relationship between gender, tumor localization, microscopic type (WHO), regional lymph node involvement, depth of invasion, N stage, TNM stage, PeNI, LVI, intensive care unit (ICU) admission, mortality, and PNI was investigated (Table 2). It was observed that there was a significant association between microscopic type (WHO), regional lymph node involvement, depth of invasion, N stage, TNM stage, ICU admission, mortality, and PNI. Specifically, patients with adenocarcinoma as the microscopic type had lower PNI values compared to those with signet ring cell carcinoma. Patients with regional lymph node involvement also had lower PNI values. Moreover, patients with T4 invasion depth had lower PNI values compared to those with T1 depth. Similarly, patients in N1 stage had lower PNI values compared to those in N0 stage, and patients in TNM stage III had lower PNI values compared to those in stage I. Patients admitted to the ICU had lower PNI values, and patients with mortality had lower PNI values.

**Table 2:** Relationship between PNI and gender, tumor localization, microscopic type (WHO), regional lymph node involvement, depth of invasion, N stage, TNM stage, PeNI, LVI, ICU admission, mortality.

		PNI		*P*
		Mean ± SD	Median (minimum–maximum)	
Sex	Male	39.98 ± 6.46	41.06 (12.21–52.61)	0.448
	Female	39.38 ± 6.13	41.01 (18.01–49.91)	
Tumor localization	Antrum	39.37 ± 7.08	40.11 (12.21–52.01)	0.544
	Cardia	40.21 ± 6.33	41.71 (12.61–51.01)	
	Corpus	39.75 ± 5.63	41.01 (26.31–52.61)	
Microscopic type (WHO)	Adenocarcinoma	39.15 ± 6.15	40.61 (12.61–51.91)	**0.002**
	Signet ring cell carcinoma	42.2 ± 5.65	42.16 (18.01–52.61)	
	Mystadenocarcinoma	41.51 ± 5.41	43.01 (35.51–46.01)	
	Mucinousadenocarcinoma	25.16 ± 18.32	25.16 (12.21–38.12)	
Regional lymph node involvement	None	41.37 ± 5.57	41.66 (26.31–52.61)	**0.018**
	Positive	39.11 ± 6.56	40.76 (12.21–51.01)	
Invasion depth	T1	42.77 ± 5.13	42.31 (26.31–52.01)	**0.004**
	T2	42.13 ± 5.58	43.62 (30.5–49.91)	
	T3	39.57 ± 7.08	41.11 (12.61–50.01)	
	T4	39.08 ± 6.28	40.11 (12.21–52.61)	
N stage	N0	41.43 ± 5.75	41.71 (26.31–52.61)	**0.010**
	N1	36.87 ± 7.52	36.11 (18.01–49.41)	
	N2	38.79 ± 5.45	40.01 (26.81–49.7)	
	N3	40.08 ± 6.44	41.21 (12.21–51.01)	
TNM stage	I	42.62 ± 5.2	43.01 (26.31–52.01)	**0.005**
	II	39.59 ± 6.34	40.91 (25.71–52.61)	
	III	39.18 ± 6.45	40.76 (12.21–51.01)	
Perineural invasion	None	40.94 ± 7.02	42.06 (12.61–51.91)	0.094
	Positive	39.61 ± 6.23	40.96 (12.21–52.61)	
Lymphovascular invasion	None	40.85 ± 8.22	41.86 (12.61–51.91)	0.128
	Positive	39.7 ± 6.17	41.01 (12.21–52.61)	
ICU admission	None	41.01 ± 5.58	41.61 (18.01–52.61)	**<0.001**
	Positive	35.47 ± 7.08	36.01 (12.21–46.01)	
Mortality	None	41.28 ± 5.27	41.61 (26.51–52.01)	**0.005**
	Positive	38.87 ± 6.8	40.76 (12.21–52.61)	

Data are presented as median (interquartile range) or n (%).

ICU = intensive care unit, LVI = lymphovascular invasion, PeNI = perineural invasion, PNI = Prognostic Nutritional Index, TNM = Tumor Node Metastasis, WHO = World Health Organization.

The values that are statistically significant (*P* < 0.05) are marked in bold.

The relationship between age, time from surgery to death, tumor size, regional lymph node count, and PNI was examined. It was found that there is an inverse correlation between age and tumor size with PNI, while there is a direct correlation between the time from surgery to death and PNI.

**Figure 1.**
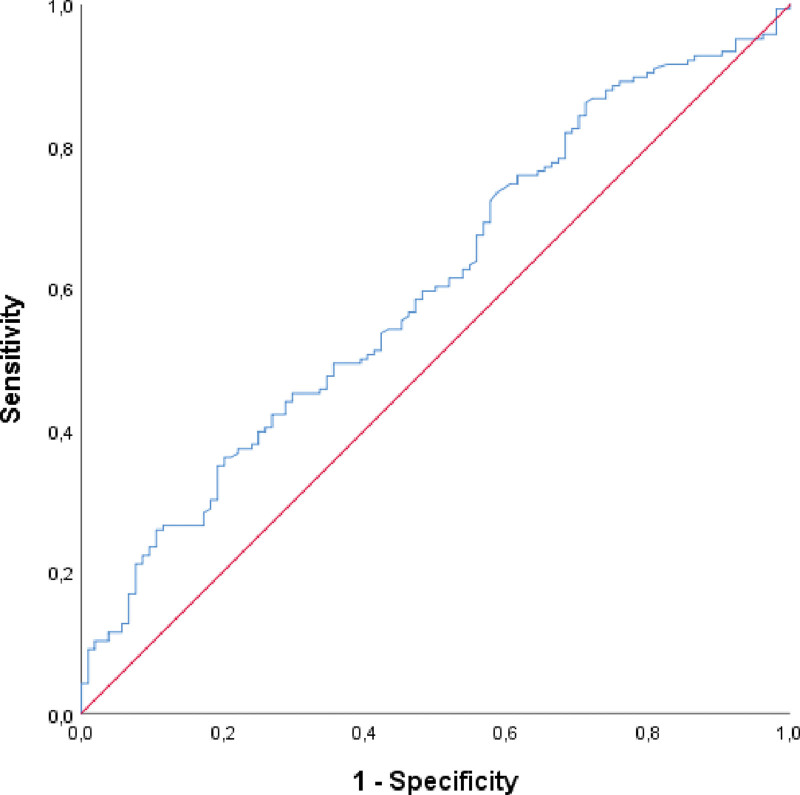
ROC curve for predicting mortality based on PNI value.

A significant cutoff value for predicting mortality using the PNI has been investigated (Table 3, Fig. 1). When a cutoff of <37 is taken for PNI, a sensitivity of 34.94%, specificity of 80.77%, positive predictive value of 74.36%, and negative predictive value of 43.75% are obtained. However, a significant cutoff value for predicting PeNI and LVI using the PNI could not be found. The regression analysis revealed that a one-unit decrease in the PNI value increased the risk of mortality by 1.068 units.

**Table 3:** Significant cutoff value at which PNI value can predict mortality.

	AUC (%95 CI)	*P*	Cutoff	Sensitivity	Specificity	PPV	NPV
Mortality	0.601 (0.533–0.670)	**.005**	PNI < 37	(34.94)	(80.77)	(74.36)	(43.75)
Perineural invasion	0.585 (0.490–0.679)	.094					
Lymphovascular invasion	0.598 (0.475–0.721)	.128					

ROC analysis. *P* value is <0.05 and therefore statistically significant.

AUC = area under curve, NPV = negative predictive value, PNI = Prognostic Nutritional Index, PPV = positive predictive value.

When patients were divided into 2 groups, the relationship between PNI and variables such as gender, tumor localization, microscopic type (WHO), regional lymph node involvement, depth of invasion, N stage, stage, PeNI, LVI, ICU admission, and mortality was examined for those in the PNI-low (PNI < 39.8) group. It was observed that in the group with PNI < 39.8, the PNI value was lower in patients with ex. There was no statistically significant relationship between PNI and lymphovascular or PeNI. In the regression analysis, it was observed that a one-unit decrease in the PNI value increased the risk of mortality by 1.138 units. In the group of patients with high PNI (>39.8), the relationship between gender, tumor localization, microscopic type (WHO), regional lymph node involvement, depth of invasion, N stage, stage, PeNI, LVI, ICU admission, mortality, and PNI was examined, and no significant results were found. The impact of PeNI on survival was examined separately for patients with PNI < 39.8 and those with PNI > 39.8. Accordingly, in the group of patients with PNI > 39.8, it was observed that those without PeNI had better survival. The regression analysis revealed that the presence of PeNI in patients with PNI > 39.8 increased the risk of mortality by 2.088 units.

The impact of LVI on survival was examined separately for patients with PNI < 39.8 and those with PNI > 39.8. It was observed that in patients with PNI > 39.8, those without LVI had better survival. In the regression analysis, the presence of LVI in patients with PNI > 39.8 increases the mortality risk by 3.171 units.

In the comparison between PNI groups, a significant relationship was observed concerning microscopic type (WHO), depth of invasion, stage, PeNI, LVI, and ICU admission. Specifically, the proportion of adenocarcinoma in the microscopic type (WHO) is higher in those with PNI < 39.8 compared to those with PNI > 39.8. The rate of T4 invasion depth is higher in those with PNI < 39.8 compared to those with PNI > 39.8. The proportion of stage III is higher in those with PNI < 39.8 compared to those with PNI > 39.8. Additionally, the rates of PeNI, LVI, and ICU admission are higher in those with PNI < 39.8 compared to those with PNI > 39.8 (Table 4). Logistic regression analysis showed that poor differentiation, T3 to 4 stage, LNM, and LVI were independent risk factors for PNI in gastric cancer.

**Table 4:** Comparison between PNI groups.

		PNI < 39.8		PNI > 39.8		*P*
		n	%	N	%	
Sex	Male	74	(66.07)	112	(70.89)	.400
	Female	38	(33.93)	46	(29.11)	
Tumor localization	Antrum	41	(36.61)	44	(27.85)	.304
	Cardia	38	(33.93)	59	(37.34)	
	Corpus	33	(29.46)	55	(34.81)	
Microscopic type (WHO)	Adenocarcinoma	91	(81.25)	110	(69.62)	**.032**
	Signet ring cell carcinoma	18	(16.07)	46	(29.11)	
	Mixed adenocarcinoma	1	(0.89)	2	(1.27)	
	Mucinous adenocarcinoma	2	(1.79)	0	(0.00)	
Regional lymph node involvement	None	28	(25.00)	54	(34.18)	.106
	Positive	84	(75.00)	104	(65.82)	
Invasion depth	T1	6	(5.36)	27	(17.09)	**.011**
	T2	5	(4.46)	12	(7.59)	
	T3	16	(14.29)	25	(15.82)	
	T4	85	(75.89)	94	(59.49)	
N stage	N0	23	(20.54)	46	(29.11)	.058
	N1	19	(16.96)	13	(8.23)	
	N2	25	(22.32)	27	(17.09)	
	N3	45	(40.18)	72	(45.57)	
TNM stage	I	10	(8.93)	33	(20.89)	**.030**
	II	20	(17.86)	25	(15.82)	
	III	82	(73.21)	100	(63.29)	
Perineural invasion	None	10	(8.93)	28	(17.72)	**.041**
	Positive	102	(91.07)	130	(82.28)	
Lymphovascular invasion	None	4	(3.57)	18	(11.39)	**.021**
	Positive	108	(96.43)	140	(88.61)	
ICU admission	None	68	(60.71)	143	(90.51)	**<.001**
	Positive	44	(39.29)	15	(9.49)	
Mortality	None	36	(32.14)	68	(43.04)	.070
	Positive	76	(67.86)	90	(56.96)	

Chi-square test. Data are presented as median (interquartile range) or n (%). *P* value is <0.05 and therefore statistically significant.

ICU = intensive care unit, PNI = Prognostic Nutritional Index, TNM = tumor node metastasis, WHO = World Health Organization.

Patients with PNI < 39.8 tend to be older. Additionally, the interval between surgery and death is shorter in patients with PNI < 39.8, while the tumor size is larger (Table 5).

**Table 5:** Relationship between PNI groups and mortality, tumor size, and lymph nodes.

	PNI < 39.8		PNI > 39.8		*P*
	Mean ± SD	Median (Min–Max)	Mean ± SD	Median (Min–Max)	
Age	66.58 ± 10.96	66.5 (26–90)	57.82 ± 11.87	59 (23–81)	**<.001**
The day between surgery and death	466.59 ± 469.69	298 (0–1886)	650.83 ± 500.83	472 (0–1984)	**.002**
Tumor size (cm)	8.52 ± 5.05	7 (1–28)	5.65 ± 3.41	5 (0–18)	**<.001**
Number of regional lymph nodes	29.15 ± 13.47	25 (16–90)	27.87 ± 10.54	25 (16–68)	.751

Mann–Whitney *U* test. *P* value is <0.05 and therefore statistically significant.

PNI = Prognostic Nutritional Index.

## 4. Discussion

GC is the 5th most common malignancy worldwide and ranks 4th in cancer-related deaths. Especially in Far Eastern countries (such as China, Korea, Japan), as well as in Russia, Iran, and Turkey, it is one of the leading causes of cancer-related deaths, and in countries where gastric cancer screening programs are not implemented, it is often diagnosed at an advanced stage.^[1]^ In recent years, PNI, LVI, and PeNI have gained importance in the treatment process of various malignancies, including GC, CRC, esophageal squamous cell carcinoma, bladder cancer, and prostate cancer, due to their effects on prognosis. GC is generally diagnosed at an advanced stage, and LNM is the main route of spread predicting a poor prognosis. However, the mechanism of LNM in GC is still not fully understood.^[1,12–19,30–32]^

PNI has been shown to be an effective prognostic factor in GC patients undergoing gastrectomy in the literature. In their study of 258 patients operated for stages 1 to 3 GC, Ishiguro et al found that low PNI value was associated with poor prognosis criteria such as tumor invasion depth, lymph node involvement, age, LVI, postoperative complications, and overall survival.^[33]^ Maejima et al from Japan investigated the relationship between PNI and postoperative complications, as well as the progression and recurrence of GC in their study consisting of 697 cases. They examined survival rates for PNI ≥ 45 (high PNI) and < 45 (low PNI) groups, and found that the 5-year overall survival (OS) and cancer-specific survival (CS) were significantly worse for the low PNI group.^[34]^ Jiang et al evaluated the clinical effects of PNI in patients undergoing total gastrectomy for GC and took a PNI cutoff value of 46 in their study. They divided the patients into low and high PNI groups and found that the 5-year OS rate in the low PNI group was significantly lower than that in the high PNI group.^[6]^ In our study, we also found that the low PNI value had a negative effect on mortality (*P* < .019), and we saw that this was consistent with the literature.

In extensive clinical studies, it has been shown that LVI in GC is associated with a high probability of LNM and is an independent factor for poor prognosis. Furthermore, it has been demonstrated that LVI worsens the prognosis of advanced GC with LNM.^[35–40]^ Fujikawa et al evaluated the relationship between LVI and clinicopathological characteristics and outcomes in their series of 2090 cases of GC, where LVI positivity was detected in 894 cases, reporting that LVI is an independent risk factor for patients’ prognosis. They also showed that the 5-year overall survival rates were lower in patients with positive LVI compared to those with negative LVI (60.9% vs 76.7%, *P* = .005).^[41]^ Blumenthaler et al, in their prospective study of 281 cases investigating the relationship between LVI and PeNI involvement in GC, found LVI in 33% of cases, PeNI in 25%, both LVI and PeNI positivity in 18%, and both negative in 61% of cases.^[42]^ Zhang et al identified LNM in 56.85% of 1488 GC patients, and found that the incidences of LVI/PeNI were 28.97%, 50.17%, 68.25%, and 83.39% in stages N0, N1, N2, and N3, respectively. They also revealed that 75.27% of patients with LVI/PeNI had LNM, and concluded that LVI/PeNI is an independent risk factor for LNM in GC.^[35]^ In our study, lymph node and LVI rates were determined to be 69.63% and 91.85%, respectively. While PNI deficiency alone had an effect on mortality, it was observed that in patients with PNI > 39.8, the presence of positive LVI was associated with worse survival (*P* < .017). Our Kaplan–Meier analysis showed a significantly different survival rate between the positive and negative LVI groups, favoring negative LVI. We observed that LVI and PeNI positivity are associated with more aggressive tumor invasion, and although the role of LVI and PeNI in the formation of LNM is still unknown, we believe that surgeries performed without adherence to oncological principles in low-volume centers are effective. Additionally, we suggest considering the suspicion of occult metastasis and close monitoring when LVI/PeNI is detected.

Clinical studies have demonstrated that PeNI-positive cases exhibit a more aggressive course, and the reported rates of PeNI positivity in the literature range from 31.7% to 65.0%.^[43–46]^ Zhao et al reported a PeNI positivity rate of 35.9% in a meta-analysis of 7004 cases. They found that in GC patients with PeNI positivity, the tumor size was larger, tumor invasion was deeper, and tumor stage was more advanced, and patients with positive PeNI had a worse survival outcome compared to those with negative PeNI.^[47]^ Chen et al reported in their retrospective study of 2752 cases that PeNI was an independent factor associated with early recurrence and worse survival after optimal curative surgery in stage I to III GC patients.^[48]^ Aurello et al found a positive PeNI rate of 45.6% in a series of 103 cases and reported a five-year overall survival rate of 42%.^[45]^ Zhang et al, in their studies investigating the effects of PNI and LVI on LNM and survival in GC and CRC, reported that the incidence of LVI/PeNI in GC was significantly higher compared to CRC, and LVI/PeNI was associated with deeper tumor invasion and more LNM, also reporting that LVI/PeNI is an independent prognostic factor in GC.^[35]^ In our study, we found that the rates of positive and negative PeNI involvement were 85.93% and 14.07%, respectively. Accordingly, it was observed that patients without PeNI had better survival in the group with high PNI (>39.8) (*P* < .025). At this point, we found that our study results were similar to the literature.

The effect of PNI on the histological and pathological characteristics of tumors has been investigated in various studies, similar to its impact on mortality. Eo et al examined the effect of PNI on survival in GC in their study, where they retrospectively analyzed 314 patients who underwent surgery for GC. They determined the PNI cutoff value as 47.3 and found no statistically significant difference in terms of PeNI between the PNI-low (<47.3) and PNI-high (>47.3) groups.^[49]^ In our study, however, when comparing the PNI-low and PNI-high groups, we found that the rates of PeNI (*P* < .041) and LVI (*P* > .021) were higher in patients with PNI < 39.8 compared to those with PNI > 39.8.

The limitations of our study include being a retrospective study. Also, the number of cases were low and the study was from a single center. Also, the adjuvant therapy is an important factor determining the overall survival and some cases operated in our center receive adjuvant treatment in other center or did not choose to receive an adjuvant therapy therefore this also created a heterogeneity in our study group.

## 5. Conclusion

In patients with GC, PNI, LVI, and PeNI are independent prognostic factors for overall survival in postoperative patients. A low PNI score is a poor prognostic factor on its own, while in patients with high PNI scores, the presence of positive LVI and PeNI adversely affects survival. We found that in patients with low PNI, the rates of PeNI and LVI are higher compared to those with high PNI, and this significantly affects mortality.

## Acknowledgments

Thank you Özgül Düzgün for statistical support and table assistance.

## Author contributions

**Conceptualization:** Pirilti Özcan, Mehmet Sinan Çarkman.

**Data curation:** Pirilti Özcan.

**Formal analysis:** Pirilti Özcan.

**Writing – original draft:** Pirilti Özcan.

**Writing – review & editing:** Mehmet Sinan Çarkman.

## References

[1] Sung H Ferlay J Siegel RL. Global cancer statistics 2020: GLOBOCAN estimates of incidence and mortality worldwide for 36 cancers in 185 countries. CA Cancer J Clin. 2021;71:209–49. 33538338 10.3322/caac.21660

[2] Ajani JA D’Amico TA Bentrem DJ. Gastric cancer, version 2.2022, NCCN clinical practice guidelines in oncology. J Natl Compr Canc Netw. 2022;20:167–92. 35130500 10.6004/jnccn.2022.0008

[3] Correa P. Gastric cancer: overview. Gastroenterol Clin North Am. 2013;42:211–7. 23639637 10.1016/j.gtc.2013.01.002 PMC3995345

[4] Smyth EC Nilsson M Grabsch HI van Grieken NC Lordick F. Gastric cancer. Lancet. 2020;396:635–48. 32861308 10.1016/S0140-6736(20)31288-5

[5] Wang FH Zhang XT Li YF. The Chinese Society of Clinical Oncology (CSCO): clinical guidelines for the diagnosis and treatment of gastric cancer, 2021. Cancer Commun (Lond). 2021;41:747–95. 34197702 10.1002/cac2.12193 PMC8360643

[6] Jiang N Deng JY Ding XW. Prognostic nutritional index predicts postoperative complications and long-term outcomes of gastric cancer. World J Gastroenterol. 2014;20:10537–44. 25132773 10.3748/wjg.v20.i30.10537 PMC4130864

[7] Migita K Takayama T Saeki K. The prognostic nutritional index predicts long-term outcomes of gastric cancer patients independent of tumor stage. Ann Surg Oncol. 2013;20:2647–54. 23463091 10.1245/s10434-013-2926-5

[8] Nozoe T Ninomiya M Maeda T Matsukuma A Nakashima H Ezaki T. Prognostic nutritional index: a tool to predict the biological aggressiveness of gastric carcinoma. Surg Today. 2010;40:440–3. 20425547 10.1007/s00595-009-4065-y

[9] Dicken BJ Graham K Hamilton SM. Lymphovascular invasion is associated with poor survival in gastric cancer: an application of gene-expression and tissue array techniques. Ann Surg. 2006;243:64–73. 16371738 10.1097/01.sla.0000194087.96582.3e PMC1449982

[10] Liebig C Ayala G Wilks JA Berger DH Albo D. Perineural invasion in cancer: a review of the literature. Cancer. 2009;115:3379–91. 19484787 10.1002/cncr.24396

[11] Skancke M Arnott SM Amdur RL Siegel RS Obias VJ Umapathi BA. Lymphovascular invasion and perineural invasion negatively impact overall survival for stage II adenocarcinoma of the colon. Dis Colon Rectum. 2019;62:181–8. 30640833 10.1097/DCR.0000000000001258

[12] Poeschl EM Pollheimer MJ Kornprat P. Perineural invasion: correlation with aggressive phenotype and independent prognostic variable in both colon and rectum cancer. J Clin Oncol. 2010;28:e358–60. 20385977 10.1200/JCO.2009.27.3581

[13] Duraker N Sişman S Can G. The significance of perineural invasion as a prognostic factor in patients with gastric carcinoma. Surg Today. 2003;33:95–100. 12616368 10.1007/s005950300020

[14] Hwang JE Hong JY Kim JE. Prognostic significance of the concomitant existence of lymphovascular and perineural invasion in locally advanced gastric cancer patients who underwent curative gastrectomy and adjuvant chemotherapy. Jpn J Clin Oncol. 2015;45:541–6. 25759484 10.1093/jjco/hyv031

[15] Wu J Chen QX. Prognostic and predictive significance of tumor length in patients with esophageal squamous cell carcinoma undergoing radical resection. BMC Cancer. 2016;16:394. 27387460 10.1186/s12885-016-2417-8 PMC4936257

[16] Muppa P Gupta S Frank I. Prognostic significance of lymphatic, vascular and perineural invasion for bladder cancer patients treated by radical cystectomy. Pathology (Phila). 2017;49:259–66. 10.1016/j.pathol.2016.12.347 28259358

[17] Zareba P Flavin R Isikbay M. Perineural invasion and risk of lethal prostate cancer. Cancer Epidemiol Biomarkers Prev. 2017;26:719–26. 28062398 10.1158/1055-9965.EPI-16-0237 PMC5413395

[18] Saeter T Vlatkovic L Waaler G. Combining lymphovascular invasion with reactive stromal grade predicts prostate cancer mortality. Prostate. 2016;76:1088–94. 27271973 10.1002/pros.23192

[19] Lu J Dai Y Xie JW. Combination of lymphovascular invasion and the AJCC TNM staging system improves prediction of prognosis in N0 stage gastric cancer: results from a highvolume institution. BMC Cancer. 2019;19:216. 30857518 10.1186/s12885-019-5416-8 PMC6413460

[20] Zhao B Huang X Zhang J. Clinicopathologic factors associated with recurrence and long-term survival in nodenegative advanced gastric cancer patients. Rev Esp Enferm Dig. 2019;111:111–20. 30404528 10.17235/reed.2018.5829/2018

[21] Kunisaki C Makino H Kimura J. Impact of lymphovascular invasion in patients with stage I gastric cancer. Surgery. 2010;147:204–11. 19878963 10.1016/j.surg.2009.08.012

[22] Bilici A Seker M Ustaalioglu BB. Prognostic significance of perineural invasion in patients with gastric cancer who underwent curative resection. Ann Surg Oncol. 2010;17:2037–44. 20333555 10.1245/s10434-010-1027-y

[23] Chiaravalli AM Cornaggia M Furlan D. The role of histological investigation in prognostic evaluation of advanced gastric cancer. Analysis of histological structure and molecular changes compared with invasive pattern and stage. Virchows Arch. 2001;439:158–69. 11561756 10.1007/s004280100441

[24] Ryu WS Kim JH Jang YJ. Expression of estrogen receptors in gastric cancer and their clinical significance. J Surg Oncol. 2012;106:456–61. 22422271 10.1002/jso.23097

[25] De Franco L Marrelli D Voglino C. Prognostic value of perineural invasion in resected gastric cancer patients according to Lauren histotype. Pathol Oncol Res. 2018;24:393–400. 28555306 10.1007/s12253-017-0257-8

[26] Kwon KJ Shim KN Song EM. Clinicopathological characteristics and prognosis of signet ring cell carcinoma of the stomach. Gastric Cancer. 2014;17:43–53. 23389081 10.1007/s10120-013-0234-1

[27] Liu E Zhong M Xu F. Impact of lymphatic vessel invasion on survival in curative resected gastric cancer. J Gastrointest Surg. 2011;15:1526–31. 21717282 10.1007/s11605-011-1600-0

[28] Kim JH Park SS Park SH. Clinical significance of immuno histo chemically-identified lymphatic and/or blood vessel tumor invasion in gastric cancer. J Surg Res. 2010;162:177–83. 20031164 10.1016/j.jss.2009.07.015

[29] Zhang F Chen H Luo D. Lymphovascular or perineural invasion is associated with lymph node metastasis and survival outcomes in patients with gastric cancer. Cancer Med. 2023;12:9401–8. 36952439 10.1002/cam4.5701 PMC10166947

[30] Yavuz A Gobut H Dikmen K Bostanci H Buyukkasap AC Yuksel O. Risk factors and clinical significances for retropancreatic lymph node metastasis in gastric cancer patients. Cir Cir. 2023;8:648–57. 10.24875/CIRU.22000270 37156166

[31] Fonseca T Coimbra M Barbosa E Barbosa J. Gastric cancer: histological response of tumor and metastatic lymph nodes for perioperative chemotherapy. Cir Cir. 2022;90:36–41. 36480751 10.24875/CIRU.21000657

[32] Rodríguez-Quintero JH Aguilar-Frasco J Morales-Maza J Sánchez-García-Ramos E Medina-Franco H Cortes-Gonzalez R. Predictors of anastomotic leak after total gastrectomy in patients with adenocarcinoma. Cir Cir. 2022;90:216–22. 35349569 10.24875/CIRU.20001220

[33] Ishiguro T Aoyama T Ju M. Prognostic nutritional index as a predictor of prognosis in postoperative patients with gastric cancer. In Vivo. 2023;37:1290–6. 37103068 10.21873/invivo.13207 PMC10188044

[34] Maejima K Taniai N Yoshida H. The prognostic nutritional index as a predictor of gastric cancer progression and recurrence. J Nippon Med Sch. 2022;89:487–93. 35644550 10.1272/jnms.JNMS.2022_89-507

[35] Zhang CD Ning FL Zeng XT Dai DQ. Lymphovascular invasion as a predictor for lymph node metastasis and a prognostic factor in gastric cancer patients under 70 years of age: a retrospective analysis. Int J Surg. 2018;53:214–20. 29609047 10.1016/j.ijsu.2018.03.073

[36] Hatta W Gotoda T Oyama T. A scoring system to stratify curability after endoscopic submucosal dissection for early gastric cancer: “eCura system.”. Am J Gastroenterol. 2017;112:874–81. 28397873 10.1038/ajg.2017.95

[37] Li P Ling YH Zhu CM. Vascular invasion as an independent predictor of poor prognosis in nonmetastatic gastric cancer after curative resection. Int J Clin Exp Pathol. 2015;8:3910–8. 26097575 PMC4466962

[38] Lee JH Kim MG Jung MS Kwon SJ. Prognostic significance of lymphovascular invasion in node-negative gastric cancer. World J Surg. 2015;39:732–9. 25376868 10.1007/s00268-014-2846-y

[39] Li P He HQ Zhu CM. The prognostic significance of lymphovascular invasion in patients with resectable gastric cancer: a large retrospective study from Southern China. BMC Cancer. 2015;15:370. 25947284 10.1186/s12885-015-1370-2 PMC4435771

[40] Yoshida M Sugino T Kusafuka K. Peritoneal dissemination in early gastric cancer: importance of the lymphatic route. Virchows Arch. 2016;469:155–61. 27220762 10.1007/s00428-016-1960-7

[41] Fujikawa H Koumori K Watanabe H. The clinical significance of lymphovascular invasion in gastric cancer. In Vivo. 2020;3:1533–9. 10.21873/invivo.11942 PMC7279808 32354959

[42] Blumenthaler AN Newhook TE Ikoma N. Concurrent lymphovascular and perineural invasion after preoperative therapy for gastric adenocarcinoma is associated with decreased survival. J Surg Oncol. 2021;123:911–22. 33400838 10.1002/jso.26367 PMC7906958

[43] Tianhang L Guoen F Jianwei B Liye M. The effect of perineural invasion on overall survival in patients with gastric carcinoma. J Gastrointest Surg. 2008;12:1263–7. 18463928 10.1007/s11605-008-0529-4

[44] Jiang N Deng JY Liu Y Ke B Liu HG Liang H. Incorporation of perineural invasion of gastric carcinoma into the 7th edition tumor-node-metastasis staging system. Tumour Biol. 2014;35:9429–36. 24972970 10.1007/s13277-014-2258-5

[45] Aurello P Berardi G Tierno SM. Influence of perineural invasion in predicting overall survival and disease-free survival in patients with locally advanced gastric cancer. Am J Surg. 2017;213:748–53. 27613269 10.1016/j.amjsurg.2016.05.022

[46] Zhou ZH Xu GF Zhang WJ Zhao HB Wu YY. Reevaluating significance of perineural invasion in gastric cancer based on double immunohistochemical staining. Arch Pathol Lab Med. 2014;138:229–34. 24476520 10.5858/arpa.2012-0669-OA

[47] Zhao B Lv W Mei D. Perineural invasion as a predictive factor for survival outcome in gastric cancer patients: a systematic review and meta-analysis. J Clin Pathol. 2020;73:544–51. 31980559 10.1136/jclinpath-2019-206372

[48] Chen YF Wang SY Le PH. Prognostic significance of perineural invasion in patients with stage II/III gastric cancer undergoing radical surgery. J Pers Med. 2022;12:962. 35743747 10.3390/jpm12060962 PMC9224547

[49] Eo WK Chang HJ Suh J. The prognostic nutritional index predicts survival and identifies aggressiveness of gastric cancer. Nutr Cancer. 2015;67:1262–9. 10.1080/01635581.2015.1082112 26583916

